# Effectiveness of a Medifast meal replacement program on weight, body composition and cardiometabolic risk factors in overweight and obese adults: a multicenter systematic retrospective chart review study

**DOI:** 10.1186/s12937-015-0062-8

**Published:** 2015-08-06

**Authors:** Christopher D. Coleman, Jessica R. Kiel, Andrea H. Mitola, Janice S. Langford, Kevin N. Davis, Linda M. Arterburn

**Affiliations:** 1 Department of Scientific and Clinical Affairs, Medifast, Inc, 11445 Cronhill Drive, Owings Mills, MD 21117 USA; 2 Independent Consultant, Clifton Park, NY 12065 USA

**Keywords:** Obesity, Weight loss, Body composition, Meal replacement, Gender, Seniors, Blood pressure

## Abstract

**Background:**

Recent medical guidelines emphasize the importance of actively treating overweight and obesity with diet and lifestyle intervention to achieve ≥5 % weight loss in a 6-month period. Commercial programs offer one approach provided there is evidence of their efficacy and safety. This study was conducted to evaluate the effectiveness of the Medifast® 4 & 2 & 1 Plan™ on weight loss, body composition and cardiometabolic risk factors in overweight and obese adults.

**Methods:**

A systematic retrospective chart review of 310 overweight and obese clients following the Medifast 4 & 2 & 1 Plan at one of 21 Medifast Weight Control Centers® was conducted. Data were recorded electronically and key data points were independently verified. The primary endpoint was change from baseline body weight at 12 weeks. Within group paired t-tests were used to examine changes from baseline in a completers population. Differences between gender and age subgroups were examined using bivariate t-tests and mixed model regression analyses.

**Results:**

For the primary endpoint at 12 weeks, body weight among completers (*n* = 185) was reduced by a mean of 10.9 ± 5.6 kg (-10.1 %, *p* < 0.0001), and at 24 weeks (*n* = 81) mean weight was reduced by 16.0 ± 7.9 kg (-14.3 %). At 12 and 24 weeks, 85 % and 96 % of those remaining on the plan, respectively, had lost ≥5 % of their baseline body weight. Lean mass was preserved to within 5 % of baseline throughout the 24 weeks, and fat mass represented ≥80 % of the body weight lost from 12 weeks onward. Men, women, seniors (≥65 years), and non-seniors (<65 years) all had significant weight reductions with preservation of lean mass. Significant improvements in blood pressure, pulse and waist-to-hip ratio were observed. Mean weight regain among the subset who entered a formal maintenance phase was <2 % during an average follow-up of 34 weeks. The meal plan was well tolerated, and program adherence was >85 %.

**Conclusions:**

The 4 & 2 & 1 Plan used at Medifast Weight Control Centers was effective for weight loss, preservation of lean mass and improvement in cardiometabolic risk factors. The plan was generally well tolerated in a broad population of overweight and obese adults. #NCT02150837.

**Electronic supplementary material:**

The online version of this article (doi:10.1186/s12937-015-0062-8) contains supplementary material, which is available to authorized users.

## Introduction

Overweight and obesity are linked to a multitude of serious comorbidities, and obesity carries an additional risk of greater all-cause and cardiovascular disease mortality [1]. Currently, 69 % of adults in the United States are overweight and 35 % are obese [2]. The health consequences of overweight and obesity have been described as “the most burdensome public health issue facing the Nation”, and both prevention and intervention are recommended [3]. While recent trends seem to indicate that overall increases in the prevalence of obesity may be leveling off, the rates of obesity in men significantly increased between 1998 and 2008, and the 2010 age-adjusted rates indicate women aged 60 and older have the highest rates of obesity at 42.3 % [4, 5]. Unfortunately, there is a paucity of research in both men and older adults as most existing weight loss intervention studies have under-represented or excluded these populations [6, 7].

For overweight or obese individuals, even a relatively small amount of weight loss can reduce the risk of developing related co-morbidities, such as cardiovascular disease, type 2 diabetes and some forms of cancers [1, 8–11]. Understanding obesity as a complex, chronic disease is essential for providing effective interventions and “obligate[s] clinicians to go beyond mere recommendations to eat less and move more” [12, 13]. Recent guidelines issued by the American Heart Association, the American College of Cardiology and The Obesity Society for the management of overweight and obesity in adults recommend participation in a comprehensive lifestyle program, which includes a reduced calorie diet along with exercise and behavior change components, as the cornerstone of all treatment options for overweight and obese individuals, with the goal of achieving clinically meaningful weight loss of at least 5–10 % in a 6-month period [1]. Commercial programs that provide a comprehensive lifestyle intervention are also supported as an option for weight loss within these guidelines, provided they are backed by evidence of their safety and efficacy. Additionally, evidence also supports the use of meal replacements as part of a structured approach to obesity treatment, as meal replacements have been shown to be a safe and effective tool for limiting calorie intake and promoting weight loss and weight maintenance among overweight and obese individuals [14, 15].

The Medifast® Program is a commercial program that features a combination of Medifast Meal Replacements, conventional food choices, and customizable levels of support for weight loss and weight maintenance. At Medifast Weight Control Centers® (MWCC), the Medifast meal plans are combined with individualized one-on-one weekly counseling to create a comprehensive lifestyle program. One of the meal plans available for weight loss is the Medifast 4 & 2 & 1 Plan™, an alternative, slightly higher calorie weight loss plan than the most frequently used Medifast 5 & 1 Plan®. The Medifast 4 & 2 & 1 Plan is often recommended for men and seniors (≥65 years), or based on specific individual behaviors or food preferences (e.g., individual engages in high levels of physical activity or wants to include fruit, dairy or whole grains daily). Both the 5 & 1 and the 4 & 2 & 1 Plans are designed to provide adequate protein to promote retention of lean mass during weight loss. Previous studies have shown the 5 & 1 Plan to be safe and effective for weight loss in overweight and obese individuals [16–18]. The purpose of this study was to evaluate the effectiveness of the Medifast 4 & 2 & 1 Plan for weight loss across 24 weeks (primary endpoint at 12 weeks) in overweight and obese adults who used this plan at MWCCs. Secondary objectives included assessing effects of this plan on body composition and cardiometabolic risk factors. *Post hoc* evaluations of body weight by gender and age category (<65 and ≥ 65 years) were also conducted.

## Methods

This study was a systematic retrospective chart review of MWCC clients who started the Medifast 4 & 2 & 1 Plan for weight loss on or after January 1, 2012 and completed the active Weight Loss phase of their program by March 31, 2014. Twenty-one MWCCs were chosen for this study based on (a) their close proximity to Medifast corporate headquarters (all MWCCs in Maryland), or (b) because they were among the centers with the largest base of clients following the 4 & 2 & 1 Plan – these MWCCs were located in Texas, Florida, and Pennsylvania. The MWCC point-of-sale system was used to identify charts of clients who purchased the 4 & 2 & 1 Plan. Once identified, charts were pre-screened at each MWCC for the presence of a signed personal health information (PHI) consent form (which included permission to use their data for research purposes), and then shipped to corporate headquarters for formal screening and data abstraction. Charts from clients who met the following study selection criteria were included: male or female overweight or obese adult (age ≥ 18 years, BMI ≥ 25 kg/m^2^), signed a PHI form, started the 4 & 2 & 1 Plan after January 1, 2012, followed the 4 & 2 & 1 Plan for at least 2 weeks, and not concurrently using any other weight loss program or pharmacotherapy for weight loss. The study was approved by an independent institutional review board (Western Institutional Review Board, Puyallup, WA) which concluded that the study met the requirements for a waiver from the informed consent process per 45 CFR 66.116(d). This study adhered to current methodological standards for retrospective chart reviews [19] and was registered in the ClinicalTrials.gov database (#NCT02150837).

The weight management program offered at the MWCC consists of weekly one-on-one in-person sessions with MWCC counselors who utilize motivational interviewing and a series of personalized behavior change strategies designed to develop behaviors that promote long-term weight management through a healthy lifestyle. MWCC counselors are trained using a combination of on-the-job and corporate-based training to ensure thorough knowledge of the Medifast products and programs and an understanding of the behavior change strategies used at MWCC. A client’s weight loss goals are determined jointly by the counselor and client, which in turn determines the prescribed length of the client’s active Weight Loss phase and overall weight management program. Programs generally include active Weight Loss, Transition, and Maintenance phases. The meal plan chosen for weight loss is also determined jointly based on a number of factors including the client’s personal preferences, lifestyle, exercise habits and medical history.

The 4 & 2 & 1 Plan is a calorie- and portion-controlled meal plan designed to stimulate gradual, steady weight loss and provides 1,100-1,300 calories daily. It consists of 4 Medifast Meal Replacements, 2 self-prepared “lean and green meals” (each including 5–7 oz of lean protein, 3 servings (~1½ cups) of non-starchy vegetables, and up to 2 healthy fat servings), and 1 healthy snack (fruit, dairy or whole grains). Medifast Meal Replacements, of which there are over 70 to choose from, each contain 90–110 calories, 11–15 g protein (primarily from soy and/or dairy), 8–15 g carbohydrates, and 0–3.5 g fat; they each share a similar nutritional profile and can be used interchangeably during the Weight Loss phase and with any of the Medifast weight loss meal plans. Following the Weight Loss phase, some MWCCs may include a Transition phase, during which calorie intake and conventional food choices are gradually increased. All individuals who meet their weight loss goal or who have completed their prescribed weight loss weeks then have the option to enter the Maintenance phase. The Medifast Maintenance Plan is based on a client’s total energy expenditure (TEE) and generally includes 3 Medifast Meal Replacements and 3 self-prepared meals (consisting of conventional food choices with serving sizes based on the Exchange List for Weight Management; the number of servings is individualized based on TEE).

Data were recorded in client charts at MWCCs by counselors. Counselors were trained to use consistent procedures when obtaining weights and anthropometric measurements. Weight was measured to the nearest 0.1 lb using a high-quality digital scale. Body composition was assessed without shoes and in light indoor clothing by direct, segmental, multi-frequency bioelectrical impedance analysis using either an InBody 230® or InBody 370® body composition analyzer (InBody Co., Cerritos, CA, USA); measurements of fat mass, percent body fat and fat free mass obtained using the InBody analyzer are highly correlated (r ≥ 0.97) to measurements made by dual energy X-ray absorptiometry (DXA) [20]. Blood pressure and pulse were measured using digital arm blood pressure monitors. Adherence was assessed based on clients’ visit attendance and self-reported meal replacement consumption.

Weight, pulse, blood pressure, and adherence-related information were abstracted at baseline and weekly throughout the client’s Weight Loss phase through 24 weeks plus at the Final Visit. The Final Visit was defined as the client’s last visit to the MWCC during active weight loss while following the 4 & 2 & 1 Plan; the time of the Final Visit varied by individual client. Anthropometrics and body composition information, which were measured approximately every 4 weeks at the MWCCs, were also collected. When available, body weight data and the corresponding dates were abstracted at the beginning and end of any other MWCC meal plans or program phases that followed a client’s use of the 4 & 2 & 1 Plan.

Notations of adverse signs, symptoms or incidents that occurred while a client was on the 4 & 2 & 1 Plan were abstracted verbatim from the chart notes, regardless of whether or not the incident appeared to be related to the intervention. This information was reviewed and categorized by a registered nurse, and simple frequencies were tabulated.

Chart data were abstracted by trained study personnel directly into electronic case report forms developed using IBM SPSS Data Collection Author and Interviewer Version 7, according to conventions developed for this study. A two-user, independent (double-data) data entry procedure was used for verification of all key data points.

### Power calculations and statistical analysis

The primary outcome in this study was change from baseline body weight at 12 weeks. Sample size was determined using a 10 % standard deviation, 0.05 significance level and with the assumption that up to 50 % of the charts would not have weight outcome data at the 12-week time point (e.g., clients completed their program, dropped out, switched to another meal plan before 12 weeks, or had missing data for this time point). From these assumptions, a minimum of 64 charts was required to attain 80 % power in order to detect clinically meaningful weight loss of 5 % from baseline using a paired *t*-test for a within-group comparison.

Data were analyzed according to a pre-defined statistical analysis plan. Normality testing was performed. Wilcoxon signed rank tests (i.e., paired t-tests for repeated measures nonparametric data) were used to compare within group changes in weight at 12 weeks compared to baseline for the primary analysis and at other predetermined time points (1, 2, 4, 8, 16, 20 and 24 weeks). When appropriate, results from parametric and non-parametric tests were performed to ensure they provided similar findings. Because of the retrospective nature of the study, predefined windows that used data closest to the specified time point were established to optimize the sample without bias: data were included if available within ±3 days for the 1 and 2 week time points, ±7 days for the 4 week time point, and within ±10 days for the remaining time points. For the primary analysis, a completers analysis was used; this analysis included each chart that had data for the given outcome and time point, irrespective of whether the individual completed his/her entire program. Similar analyses were conducted on secondary outcomes. An intention-to-treat (ITT) last observation carried forward (LOCF) analysis, pre-specified in the protocol, was also performed for the primary outcome for comparison. If missing, imputed data were carried through from the last measured observation to each client’s last prescribed week of weight loss. Additionally, in order to maximize the use of all data, including those with missing data, a pre-specified mixed model regression approach was used on the primary outcome, weight, with time as the independent variable and baseline weight as a covariate. The proportions of individuals achieving ≥5 % and ≥10 % weight loss from baseline were calculated. *Post hoc* subgroup analyses by gender and by senior status (<65, ≥65 years) were performed on body weight and body composition. Weight results were converted from lbs to kg and circumference measurements were converted from inches to cm. Program adherence was defined for meal replacements as: ≥75 % compliance of meal replacement consumption (i.e., on average 3 of the 4 assigned meal replacements were reported consumed) and for attendance as reporting to ≥75 % of their weekly visits while on the program. Differences between groups were examined using bivariate t-tests as well as mixed model regression analyses. Significance was defined as *p* < 0.05 with no adjustments for multiplicity. Analyses were conducted using SPSS Version 14.0 and Stata Version 10.

## Results

### Chart selection and flow

**Fig. 1:**
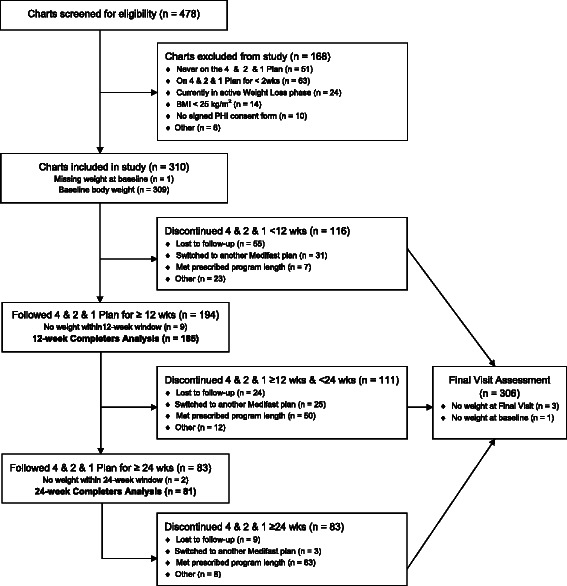
Flow diagram. Chart disposition at week 12 (primary endpoint), week 24 and Final Visit. The Completers population included all individuals that had weight data within the specified visit window. Final Visit represents an individual’s last visit to the MWCC while following the 4 & 2 & 1 Plan. The time of the Final Visit varies by individual, depending on when they discontinued the 4 & 2 & 1 Plan

Of the 478 charts received for screening, 310 met the study entry criteria and were included in the study. Five criteria accounted for 96 % of the charts excluded during the screening process (Fig. 1). Sixty percent (*n* = 185) of individuals who started the 4 & 2 & 1 Plan remained on this plan and had weight data within the 12-week visit window and were, therefore, included in the completers analysis at the 12-week primary endpoint; 26 % (*n* = 81) remained on the 4 & 2 & 1 Plan and had weight data within the 24-week visit window and were, therefore, included in the completers analysis at this time point. Among the main reasons for discontinuing the 4 & 2 & 1 Plan were loss to follow-up, switching to another Medifast meal plan, and completion of the individual’s prescribed program length (Fig. 1). The Intention-to-Treat Last Observation Carried Forward (ITT LOCF) analyses (not shown in the diagram) included all charts with baseline weight data and for which the prescribed program length did not exceed the specified time point. The ITT LOCF analysis at 12 weeks included 281 charts (28 charts were excluded because the prescribed program length was <12 weeks, and one was excluded for no baseline weight); the ITT analysis at 24 weeks included 157 charts (excluded were 151 because the prescribed program length was <24 weeks, one because a prescribed program length was not specified, and one for no baseline weight).

### Baseline characteristics

The group was comprised of 57.1 % females and 42.9 % males (Table 1). The mean (±SD) BMI was 37.7 ± 6.8 kg/m^2^ and included a broad distribution of weight classes including overweight, and class I, II and III obesity. The mean age was 53.5 ± 14.7 years, and 28 % were seniors age 65 years or older. Self-reported co-morbid conditions were prevalent in the group, including high blood sugar/diabetes (predominantly type 2), high blood pressure, and arthritis.

**Table 1:** Baseline demographics

Demographic characteristic	Mean ± SD or n (%)
N	310
Gender	
Female	177 (57.1 %)
Male	133 (42.9 %)
Age (yrs)	53.5 ± 14.7
Seniors (age ≥ 65 years)	88 (28.4 %)
Weight^a^ (kg)	109.1 ± 21.7
BMI^b^ (kg/m^2^)	37.7 ± 6.8
BMI category^b^	
Overweight (BMI ≥ 25 & <30 kg/m^2^)	32 (10.4 %)
Class I obesity (BMI ≥ 30 & <35 kg/m^2^)	87 (28.3 %)
Class II obesity (BMI ≥ 35 & <40 kg/m^2^)	78 (25.4 %)
Class III obesity (BMI ≥ 40 kg/m^2^)	110 (35.8 %)
Lean body mass^c^ (kg)	61.8 ± 14.2
Body fat mass^c^ (kg)	47.0 ± 13.9
Current smoker	19 (6.1 %)
Co-morbid conditions	
Diabetes//high blood sugar	65 (21.0 %)
High blood pressure	130 (41.9 %)
Arthritis^d^	57 (23.7 %)
Heart disease or past heart attack	14 (4.5 %)
Kidney disease	10 (3.2 %)
Liver disease	4 (1.3 %)

Abbreviations: *BMI* Body Mass Index

^a^*n* = 309; ^b^*n* = 307; ^c^*n* = 286; ^d^*n =*241

### Body weight

**Fig. 2:**
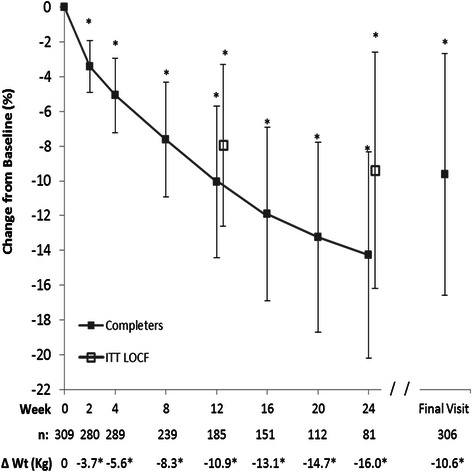
Percent change from baseline body weight. Mean (±SD) for the Completers population which included all individuals with weight data at the given visit; sample sizes are designated below the graph. Intention-to-Treat Last Observation Carried Forward (ITT LOCF) values are also shown for 12 and 24-week visits. Final Visit represents an individual’s last visit to the MWCC while on the 4 & 2 & 1 Plan. Absolute weight changes in kg are shown below the graph. Within group changes from baseline body weight using Wilcoxon signed-rank tests are shown: * *p* < 0.0001

For the primary endpoint at 12 weeks, weight among completers (*n* = 185) was reduced by a mean of 10.9 ± 5.6 kg (−10.1 %, *p* < 0.0001, Fig. 2). Overall, mean body weight decreased steadily and significantly compared to baseline through 24 weeks and at Final Visit among completers (*p* < 0.0001 for all time points; Fig. 2 and Additional file 1). The most rapid decrease occurred in the first 2 weeks (mean ± SD: −3.7 ± 1.9 kg or −3.4 % of baseline weight, *n* = 280). Among the 81 individuals with weight data at 24 weeks, all had lost weight (−16.0 ± 7.9 kg or −14.3 % of baseline weight). In a random effects regression model, controlling for baseline weight, the average rates of weight loss (95 % CI) over 2, 12, and 24 weeks were −1.89 (−1.99, −1.79), −0.84 (−0.86, −0.82), and −0.63 (−0.65, −0.61) kg per week (*p* < 0.0001, see Additional file 2). As previously noted, Final Visit describes the last weekly visit each client attended during active weight loss while following the 4 & 2 & 1 Plan, and the time to Final Visit varied by individual client. Mean weight loss at the clients’ Final Visit on the 4 & 2 & 1 Plan (*n* = 306, mean length of participation 17.4 weeks) was -10.6 ± 8.5 kg corresponding to a 9.6 % reduction in baseline weight. In agreement with the completers analysis, the results of the ITT LOCF analysis also showed steady and significant (*p* < 0.0001) weight reductions at all times, with 8.9 kg (7.9 %) and 11.5 kg (9.4 %) reductions at 12 and 24 weeks, respectively (see Fig. 2 and Additional file 3).

**Fig. 3:**
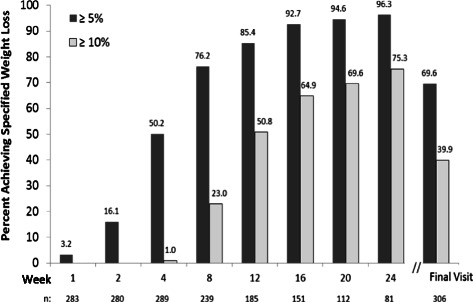
Proportion of individuals with at least 5 % and at least 10 % reduction in baseline body weight. Analysis of the Completers population which included all individuals with weight data at the given visit; sample sizes are designated below the graph. Final Visit represents an individual’s last visit to the MWCC while on the 4 & 2 & 1 Plan

In the completers analysis, half (50.2 %) of the individuals had lost at least 5 % of their baseline body weight by 4 weeks (Fig. 3). Of the clients who followed the program for at least 12 weeks, 85 % had lost at least 5 % and half had lost at least 10 % of their baseline body weight. By 24 weeks, nearly all (96 %) had lost at least 5 % and three quarters had lost at least 10 % of their baseline body weight. In an ITT LOCF analyses, approximately 70 % had lost ≥5 % of their baseline weight by 12 and 24 weeks (Additional file 4).

### Body composition

**Fig. 4:**
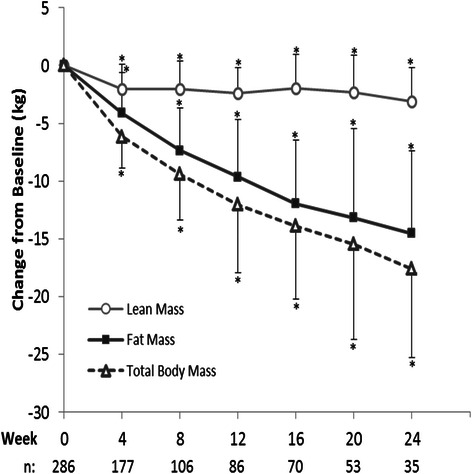
Change from baseline body weight, lean body mass and body fat mass. Mean (±SD) for the Completers population which included all individuals with weight data at the given visit; sample sizes are designated below the graph. Within group changes from baseline using Wilcoxon signed-rank tests are shown: * *p* < 0.0001

Weight loss on the 4 & 2 & 1 Plan was accompanied by significant reductions in body fat mass and proportionate increases in lean body mass: lean body mass increased from 57 % of body weight at baseline to 62 % at 12 weeks and to 64 % at 24 weeks, whereas body fat mass decreased from 43 % of body weight at baseline to 38 % at 12 weeks and to 35 % at 24 weeks. Absolute lean mass decreased during the first 4 weeks on the plan (−2.0 ± 2.1 kg, *p* < 0.0001) and was largely conserved thereafter (3.1 ± 2.9 kg reduction at 24 weeks, *p* < 0.0001, representing <5 % of baseline lean mass) compared to body fat mass which declined continuously throughout the study period (Fig. 4). Fat mass was ultimately reduced by 14.5 ± 7.2 kg, representing >30 % reduction from baseline at 24 weeks. Fat mass represented approximately 67, 80 and 83 % of the total body mass lost at 4, 12 and 24 weeks, respectively.

### Subgroup analysis of body weight and composition by gender and senior status

**Fig. 5:**
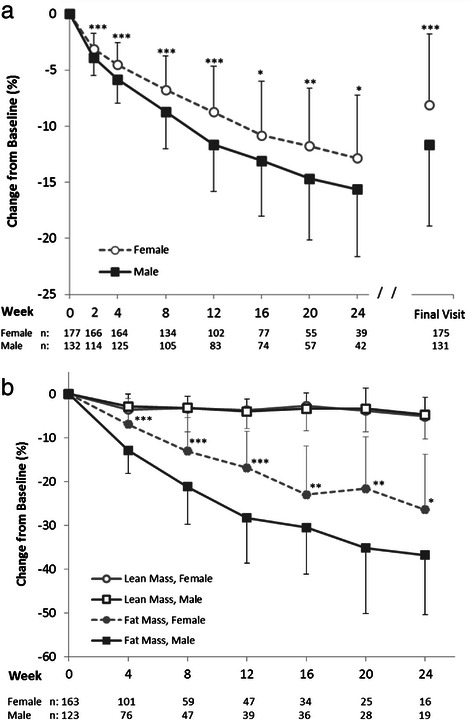
Percent change from baseline in (**a**) body weight and (**b**) lean and fat mass by gender. Mean (±SD) for the Completers population which included all individuals with weight data at the given visit; sample sizes are designated below the graph. Final Visit represents an individual’s last visit to the MWCC while on the 4 & 2 & 1 Plan. Significance levels for all within group changes from baseline were *p* < 0.0001 for body weight and fat mass and *p* < 0.01 lean mass (not shown). Significance levels for between group comparisons using bivariate t-tests at each time point are shown: *p < 0.05; **p < 0.01; ***p < 0.0001

Weight loss was first examined by gender. The mean baseline body weight of females (102.2 ± 21.1 kg, *n* = 177) was lower (*p* < 0.0001) than males (118.5 ± 19.1 kg, *n* = 132). Both males and females had significant reductions (within group *p* ≤ 0.001) in body weight through 24 weeks. Bivariate t-tests revealed that men had significantly greater reductions in absolute (*p* ≤ 0.001, Additional file 5) and percent (*p* < 0.05, Fig. 5a) body weight compared to women at all times. This finding was confirmed by a random effects regression model, controlling for baseline weight and time, which showed a main effect of gender: males lost on average 1.4 kg more than females through the first 12 weeks (*p* < 0.0001). Body composition was also examined by gender. Both males and females had considerable reductions from baseline in body fat mass (-36.8 % for males and −26.4 % for females) and smaller reductions (up to approximately 5 % of baseline) in lean body mass (*p* < 0.01 for within group analyses at all times, Fig. 5b). Bivariate analyses revealed that males lost a significantly higher percentage of their baseline fat mass (*p* < 0.05), but not significantly more of their baseline lean mass, than females. Similarly, in absolute terms, with the exception of weeks 16 and 24, males lost significantly more fat mass, and other than week 12, not more lean mass than females (Additional file 5).

**Fig. 6:**
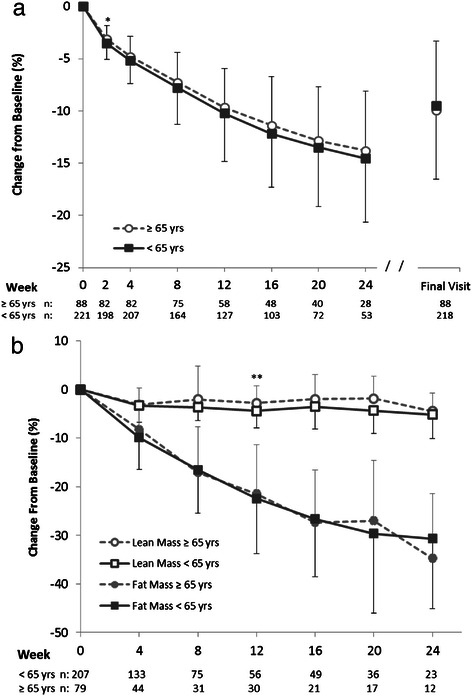
Percent change from baseline (**a**) body weight and (**b**) lean and fat mass by age group. Mean (±SD) for the Completers population grouped by age (<65 and ≥65 years). Completers included all individuals with weight data at the given visit; sample sizes are designated below the graph. Final Visit represents an individual’s last visit to the MWCC while on the 4 & 2 & 1 Plan. Significance levels for all within group changes from baseline were *p* < 0.0001 for body weight, *p* ≤ 0.002 for fat mass and *p* ≤ 0.05 for lean mass (not shown). Significance levels for between group comparisons using bivariate t-tests at each time point are shown: *p < 0.05; **p < 0.01; ***p < 0.0001

Since this plan is often used by seniors (age ≥65 years), weight loss was further examined by age. The mean baseline body weight for non-seniors (<65 years, *n* = 221, 113.7 ± 21.3 kg) was higher (*p* < 0.0001) than for seniors (97.5 ± 18.5 kg, *n* = 88). Both seniors and non-seniors had significant reductions in body weight throughout 24 weeks and at Final Visit (*p* < 0.0001 compared to baseline). Bivariate t-tests showed although non-seniors lost more absolute weight than seniors (*p* < 0.05 through 20 weeks, Additional file 6), no differences in the percentage of weight lost between the age groups was observed, except at 2 weeks (*p* = 0.039, Fig. 6a). In a random effects regression model, controlling for baseline weight and time, there was not a main effect of age (i.e., seniors vs. non-seniors) on weight lost at 12 weeks; however, when also controlling for gender, the relationship between senior status and weight change became statistically significant. We then examined for and found a significant interaction between gender and senior status (*p* < 0.05). The rate of body weight change through the primary time point of 12 weeks was therefore examined for each gender/senior subgroup: non-senior males had the highest rate of weight loss (−1.06 kg/week; −1.15,−1.06 95 % CI), followed by senior males (−0.89 kg/week; −0.94,−0.84), non-senior females (−0.72 kg/week; −0.75,−0.69) and finally senior females (−0.59 kg/week; −0.62,−0.56); all groups had significant rates of weight loss (*p* < 0.0001).

Body composition was also examined by age group. Both seniors and non-seniors had large, significant (*p* < 0.01) within group reductions in body fat mass at each time point, peaking at over 30 % loss of fat mass at 24 weeks. Lean body mass also declined (*p* < 0.05) within each group at each time point, but to a much lesser extent than did fat mass (peak loss of approximately 5 % from baseline at 24 weeks). Percent changes from baseline in both lean and fat mass were similar between groups, with only small, sporadic differences noted (Fig. 6b).

### Cardiometabolic risk factors

Based on measured baseline blood pressure (*n* = 198), 38 % were prehypertensive (systolic of 120–139 mmHg and/or diastolic of 80–89 mmHg) and 48 % were hypertensive (systolic ≥140 mmHg and/or diastolic ≥90 mmHg). A reduction in both systolic and diastolic blood pressure and heart rate occurred early in weight loss, with the majority of the response occurring within the first 4 weeks (Table 2). Average reductions in blood pressure were 11.3 ± 16.7 mm Hg and 6.6 ± 12.6 mm Hg for systolic and diastolic blood pressure, respectively, at 12 weeks (*p* < 0.0001). Improvements in blood pressure were reflected in favorable shifts in individual blood pressure categories (from hypertensive to prehypertensive or from prehypertensive to normal) in 47, 49 and 42 % of the individuals on this plan at 4, 12 and 24 weeks, respectively. Heart rates decreased, on average, by 3.0 and 3.7 beats per minute at 12 and 24 weeks, respectively (*p* < 0.05). Waist and hip circumferences were reduced (*p* < 0.0001) at all times throughout the study (Table 2), with significant corresponding improvements in waist-to-hip ratio through 12 weeks (*p* < 0.01). BMI declined (*p* < 0.0001) at all time points.

**Table 2:** Cardiometabolic risk factors

	Mean (SD)											
	4 weeks			12 weeks			24 weeks			Final visit		
	n	Baseline	Change	n	Baseline	Change	n	Baseline	Change	n	Baseline	Change
BMI (kg/m^2^)	287	37.5 (6.3)	−1.9^***^ (0.9)	185	37.0 (6.2)	−3.7^***^ (1.7)	81	38.2 (6.0)	−5.5^***^ (2.6)	304	37.6 (6.8)	−3.6^***^ (2.8)
SBP (mmHg)	175	130.5 (16.1)	−9.9^***^ (16.9)	114	129.5 (15.9)	−11.3^***^ (16.7)	52	130.2 (16.9)	−7.8^**^ (19.4)	162	131.3 (16.5)	−9.5^***^ (15.6)
DBP (mmHg)	175	86.7 (11.1)	−5.9^***^ (12.1)	114	86.1 (10.4)	−6.6^***^ (12.6)	52	85.8 (11.9)	−3.9^*^ (14.2)	163	86.7 (11.4)	−5.6^***^ (11.6)
Pulse (BPM)	175	73.4 (12.0)	−2.9^***^ (10.1)	111	72.7 (11.2)	−3.0^***^ (10.5)	51	74.2 (11.3)	−3.7^*^ (9.3)	161	74.2 (12.2)	−2.3^**^ (11.6)
WC (cm)	158	112.9 (14.8)	−5.0^***^ (3.5)	73	111.0 (14.1)	−9.8^***^ (5.9)	24	114.9 (16.7)	−13.6^***^ (7.3)			
HC (cm)	158	124.8 (13.6)	−4.0^***^ (3.5)	73	123.5 (13.6)	−8.7^***^ (5.6)	24	125.2 (12.7)	−12.5^***^ (6.8)			
WHR	158	0.91 (0.10)	−0.01^***^ (0.04)	73	0.90 (0.09)	−0.02^**^ (0.05)	24	0.92 (0.11)	−0.02 (0.05)			

Abbreviations: *BMI* Body Mass Index, *SBP* Systolic Blood Pressure, *DBP* Diastolic Blood Pressure, *BPM* Beats Per Minute, *WC* Waist Circumference, *HC* Hip Circumference, *WHR* Waist-to-Hip Ratio. Within group changes from baseline using Wilcoxon signed-rank tests: ^*^ *p* < 0.05; ^**^ *p* < 0.01; ^***^ *p* < 0.0001

### Program adherence

The average number of meal replacements consumed on the 4 & 2 & 1 Plan based on self-report, was ≥ 3.4 of the 4 assigned per day, corresponding to >85 % adherence through 24 weeks. The adherent group (see definition in methods; *n* = 259) lost more weight during weeks 8 through 24 and at Final Visit compared to the non-adherent group (*n* = 50, *p* < 0.05). When meal replacement adherence was considered only through week 12, the adherent group (*n* = 260) had greater weight loss at 8 weeks, 12 weeks, and Final Visit (*p* < 0.05), than the non-adherent group (*n* = 49). Adherence to weekly visits was also examined: on average, clients attended 85 % of their weekly counseling sessions while on the program. When examined with bivariate analyses comparing adherent and non-adherent groups, no relationship was found between attendance at weekly MWCC counseling sessions and weight loss on this plan.

### Other program information

Individuals on the 4 & 2 & 1 Plan were prescribed an average of 26.2 weeks of weight loss, and stayed on the 4 & 2 & 1 Plan for an average of 17.4 weeks. Twenty one percent (65/310) who started active weight loss on the 4 & 2 & 1 Plan subsequently changed to another Medifast weight loss meal plan(s) and spent an average of 16.0 additional weeks in active weight loss. During this time, they lost, on average, an additional 2.6 kg or 2.7 % of baseline body weight, for a total loss of approximately 12 kg over the entire weight loss period (4 & 2 & 1 and all other Medifast weight loss plans).

Following the Weight Loss phase, 43 of 310 (14 %) individuals on the 4 & 2 & 1 Plan entered the Transition phase for an average of just under 4 weeks during which individuals continued to lose approximately 0.5 kg, <1 % of baseline body weight (*p* = 0.020). Sixty-two of 310 (20 %) individuals entered the Maintenance phase for an average of 34 weeks during which individuals regained, on average, 1.6 kg (*p* < 0.0001, 1.9 % of weight at start of this phase). Despite this regain, the weight of these individuals remained 16.8 ± 10.2 kg (15–16 %) below their baseline body weight (*p* < 0.0001). Those individuals who started the Maintenance Phase had lost, on average, significantly (*p* < 0.0001) more weight (−16.2 ± 8.6 kg) specifically while on the 4 & 2 & 1 Plan compared to those who never started the Maintenance phase (−9.2 ± 7.8 kg, *n* = 244).

### Safety

Signs, symptoms and incidents notated in the charts represented lay accounts, and their descriptions were typically quite general in nature; relatedness and severity often could not be assessed from the information provided. Focusing on those that occurred at frequencies of >5 %, signs and symptoms were primarily gastrointestinal disturbances (10.6 % constipation, 7.1 % abdominal gas/bloating) or general complaints of hunger (18.1 %), fatigue (11.0 %), and stress/anxiety (5.2 %), or generally feeling sick (22.3 %) at some time during the period of study. This latter term was too general to categorize or assess further. High blood pressure (not necessarily worsening) was noted in 6.5 % of charts; a majority of these reports occurred in individuals who had pre-existing hypertension. There were 9 reports (2.9 %) of serious events, including stroke, heart attack, two cholecystectomies, and hospitalizations associated with a blood clot, ruptured hemorrhoid, high fever, and for two unspecified reasons. The ruptured hemorrhoid was secondary to constipation. Four of 310 clients (1.3 %) reported gall bladder pain/stones, including the two noted above which required surgery.

## Discussion

The use of portion-controlled meal replacements as a part of a structured meal plan has been shown to be a safe and effective method for increasing dietary compliance and providing clinically meaningful, sustainable weight loss and improvements in weight-related disease risk factors [14, 16, 17, 21–23]. This retrospective chart review study sought to characterize the effectiveness of the Medifast 4 & 2 & 1 Plan using a systematic approach in a broad sample of real world customers who used the plan in the setting of an MWCC. MWCC weight loss programs include weekly visits with behavioral counseling. This study demonstrated the program was effective for producing steady, significant weight loss over 24 weeks across a broad population of adults who were overweight or obese. At the primary endpoint of 12 weeks, mean weight loss from baseline among completers of −10.9 kg (-10.1 %) compares favorably with results from a meta-analysis of low calorie (>800 to ≤1600 kcal/day) meal replacement studies, in which the average pooled weight loss of completers at 3 months ranged between 6.19 and 6.50 kg (approximately 7 % from baseline) [14]. Differences between these results, however, may be due to differences in the study populations and/or the wider calorie range of the studies included in the meta-analysis. Regardless, half of those on the 4 & 2 & 1 Plan had clinically meaningful weight loss of 5 % or more from baseline starting as early as 4 weeks, 85 % had lost ≥5 % by the 12-week primary endpoint. Nearly all individuals (96 %) who stayed on the 4 & 2 & 1 Plan for 24 weeks had lost at least 5 % and three quarters had lost at least 10 % of their baseline body weight, thus meeting guidance goals [1]. In this study, Final Visit (clients’ last weekly visit while following the 4 & 2 & 1 Plan) represents a more stringent assessment of the effectiveness of the plan because it takes into consideration attrition that took place before 24 weeks. This analysis found that approximately 70 % had lost ≥5 % of their baseline body weight while on the program, irrespective of how long they stayed on the plan. This latter finding was similar to the ITT LOCF results.

Most weight loss studies have focused primarily on women [6], and relatively few studies include adults over the age of 65 years [7]. The population in this study reflected the demographics of customers who used the 4 & 2 & 1 Plan at MWCCs, and included 43 % men and 28 % seniors age ≥65 years. Subgroup analyses showed both men and women, and seniors and non-seniors had significant weight loss through 24 weeks and at Final Visit. Similar to other studies, after controlling for baseline weight, men lost more weight than women in both age categories [24]. One explanation may be the set calorie level provided by the plan, which would generally represent a greater caloric deficit for men who, as a group, weighed more than women at baseline. However, other physiological and/or behavioral factors known to contribute to differences in weight loss between the genders may also have factored into this differential response [24, 25].

Across the entire study population, weight loss on the 4 & 2 & 1 Plan was primarily a result of reductions in body fat mass (≥80 % of the weight lost was from fat starting from 12 weeks onward), accompanied by much smaller losses in lean mass, the latter of which occurred early in the weight loss phase and then stabilized. Although some loss of lean mass is always expected during weight loss [26], minimizing lean mass loss is an important health and safety consideration in order to maintain strength and physical function and also to maximize basal metabolic rate for long term weight maintenance. At 4 weeks, approximately 33 % of the total weight change was due to loss of lean mass, whereas at later time points, lean mass represented considerably smaller proportions (<20 %) of the total weight lost. Although the changes in the proportion of lean to total body weight during energy deficit can vary considerably depending on many factors (e.g., protein intake, activity type and level, stage of weight loss, gender, BMI, age), cross-sectional estimates of these proportions typically range from 36 to 40 % for men and from 31 to 33 % for women [26–29]. This program fared well against these estimates. While increases in activity were encouraged, the program did not include a structured exercise regimen; therefore, the nutritional composition of this meal plan is likely an important contributing factor in the retention of lean mass. Recent research suggests protein intake in the range of at least 1.1 to 1.6 g/kg body weight is optimal for retention of lean mass during weight loss [30–32]. The 4 & 2 & 1 Plan provides approximately 120–160 g/day of high quality protein which roughly translated into approximately 1.1 to 1.5 g protein/kg body weight (assuming mean baseline weight of 109.1 kg) in this study population.

Conservation of lean mass is a particularly important safety consideration in seniors due to naturally-occurring sarcopenia and concerns over loss of muscle mass and possible concomitant loss in physical function [7]. For this reason, body composition between seniors and non-seniors was compared. To account for the fact that the non-seniors group weighed more at baseline, lean and fat masses were examined as a percentage of their respective baseline values. This analysis showed seniors and non-seniors had nearly identical changes in lean and fat mass. Both groups lost more than 30 % of their baseline fat mass, and neither group lost more than 5 % of their initial lean mass on the program. Thus seniors and non-seniors alike had a strong tendency to conserve lean body mass while losing fat mass on the 4 & 2 & 1 Plan. Indeed, a recent 12-week pilot study in older adults who used four Medifast Meal Replacements daily reported 7.8 kg reduction in body weight (comprised of 5.3 kg fat mass and 2.5 kg lean mass) with no loss in physical function [33].

Body composition was also examined by gender as previous reports have suggested that changes in body composition during energy deficit may be gender specific [27, 34]. Males lost significantly more fat mass (absolute and as a proportion of baseline fat mass) but similar absolute and proportional lean mass compared to females. Thus it is reasonable to conclude that the larger weight loss experienced by males was due primarily to greater losses of fat mass, and that the 4 & 2 & 1 Plan was equally efficient at preserving lean mass in both genders.

An important medical goal of intentional weight loss is to reduce cardiovascular risk factors. Reductions in BMI, blood pressure (systolic and diastolic), heart rate, and central adiposity all accompanied weight loss on the 4 & 2 & 1 Plan. The reduction in both systolic and diastolic blood pressure and heart rate occurred early in weight loss, with the majority of the response occurring within the first 4 weeks. During this time, individuals lost an average of 5 % of baseline body weight, reinforcing the tenet that even modest reductions in weight lead to clinically meaningful health benefits. The magnitude of the blood pressure reductions observed in this study (10–11 mm Hg systolic and 4–6 mm Hg diastolic) is associated with a substantial reduction in cardiovascular disease risk [35].

Adherence is generally a key factor in weight loss success [36, 37]. Results from previous studies have shown that the use of highly structured meal plans [38, 39], consumption of portion-controlled meal replacements [14, 21, 23, 36], and support session attendance [16, 36, 37] may promote greater adherence and are all positively associated with improved weight loss outcomes. In this study, program adherence was assessed based on self-reported consumption of meal replacements and by attendance at weekly visits. Both of these program adherence components were high (>85 %) while individuals were on the program. Adherence to meal replacement consumption was positively related to weight loss, whereas an association with visit attendance was not detected in this study. In a previous chart review of clients following the Medifast 5 & 1 Plan at MWCCs, adherence (both meal replacement and attendance) was approximately 70 %, and both factors were positively related to weight loss [16]. Despite differences in data collection and analysis methods, meal replacement adherence was significantly associated with weight loss in both studies, reinforcing the importance of this factor to weight loss success on these programs. The higher overall rate of attendance adherence in the current study may have made an association more difficult to detect than in the previous study.

A limitation of the study was that data for the primary endpoint, change in body weight at 12 weeks, was only available for approximately 60 % of the study sample. Given the retrospective nature of this study, data missing at a specified time point may not be a true measure of attrition *per se*, as factors such as completion of prescribed program length, no visit/data within the window of that specific time point, and changing to another weight loss meal plan might not be considered attrition in a traditional sense. This may be an important consideration given that more than 20 % of charts indicate the reason for a client’s Final Visit on the 4 & 2 & 1 Plan was to switch meal plans and continue their weight loss on another Medifast plan(s). Nonetheless, retention of approximately 60 % at 12 weeks is generally lower than that the rates previously reported in a meta-analysis of randomized, controlled weight loss trials, but higher than other commercial programs where reported retention at 10 weeks and 3 months was 50 and 42 %, respectively [40, 41]. To address the concern of missing data, data for the primary outcome were analyzed by three different methods (completers, ITT LOCF and mixed model regression analyses); all methods provided consistent results. Another observation in this study was the relatively large variability in responses. This is similar to other retrospective weight loss studies [16, 40] and likely reflects the fact that the study included real customers who were not following a defined study protocol. Moreover, the study population came from over 20 centers and was highly heterogeneous as a result of the broad entrance criteria. These latter points support the generalizability of the study results among MWCC clients and provide a true picture of customer experience on this program. Nonetheless, this study was retrospective in nature, and as such, was neither randomized nor controlled. Evaluation of the 4 & 2 & 1 Plan in a prospective, randomized, controlled study would provide a broader assessment of efficacy.

The retrospective nature of this study limits any weight data available after use of the 4 & 2 & 1 Plan to those who subsequently followed another Medifast plan. Accordingly, weight maintenance data in this study are limited to the relatively small proportion of individuals (20 %, *n* = 62) who entered a formal Maintenance phase; however, the available data indicated minimal weight regain (average of <2 kg, <2 %) during an average follow-up period of 34 weeks. The total follow-up period for this group, including the Weight Loss, Transition (as applicable) and Maintenance phases, was over a year, with overall sustained weight loss of 17 kg. Weight at the end of the Maintenance phase remained 15–16 % below the group’s baseline weight, suggesting the program was effective for weight maintenance for those who entered this phase.

All recorded medical events were collected and analyzed in a systematic fashion. An evaluation of these events was consistent with previously reported side effects [16], primarily gastrointestinal disturbances, and general complaints of hunger, fatigue, and stress which are also often associated with intentional weight loss. Both obesity and weight loss are known to be significant risk factors for the development of gallstones [42, 43]. Just over 1 % of individuals reported gallbladder pain/stones, some requiring surgery. This rate is considerably lower than the 10–36 % rate of gallstones reported among obese subjects who lose weight rapidly [43–46], perhaps reflecting a more moderate rate of weight loss and adequate fat provided by the meal plan. In contrast to most prospective weight loss studies that limit the study population to obese but generally healthy individuals, this study population had very broad inclusion criteria and included many individuals with baseline health issues, some serious (e.g., diabetes, kidney, liver and heart disease). Given the high prevalence of pre-existing health issues and the relatively long study period (6 months), the number of serious medical issues reported was relatively low. Indeed, the observed rate of serious events is similar to placebo rates observed in pharmacotherapy trials with similar populations [47–49]. Although the safety data were limited by the retrospective nature of the study, based on the available data, the 4 & 2 & 1 Plan appeared to be generally well tolerated in this broad range of people with various health issues, and may have possible lower than expected rates of gallbladder issues.

This study reinforces previous findings that weight loss programs incorporating meal replacements can be an effective approach to weight loss and weight maintenance [14, 21]. Another Medifast meal replacement plan, the 5 & 1 Plan, has also been shown to be effective for weight loss in both retrospective studies and controlled trials [16–18].

## Conclusions

The Medifast 4 & 2 & 1 Plan used in a structured, supportive environment of a commercial weight control center was effective over a 6-month period for achieving clinically meaningful weight loss and preserving lean body mass in a broad population of overweight and obese adults. Among the subset of those who entered the Maintenance phase, the Medifast Maintenance Plan was also effective for maintaining weight loss to within 2 % of the start of this phase. Overall, the Medifast program appeared to be well tolerated. While randomized clinical trials are needed to broaden these study results, this retrospective analysis demonstrates that when used in the setting of an MWCC, the 4 & 2 & 1 Plan meets medical goals for weight loss with concomitant improvements in cardiovascular risk markers. Thus, the 4 & 2 & 1 Plan would be an appropriate commercial choice for individuals or as an option for clinician referrals.

## Additional files

Additional file 1: **Absolute and Percent Weight Change - 4 & 2 & 1 Plan – Completers Analysis.** (PDF 38 kb) Additional file 2: **Average Weekly Rate of Weight Loss by Time – 4 & 2 &1 Plan.** (PDF 51 kb) Additional file 3: **Body Weight – Intention-to-Treat Last Observation Carried Forward - 4 & 2 & 1.** (PDF 42 kb) Additional file 4: **Percent Achieving Specified Weight Loss – ITT LOCF.** (PDF 73 kb) Additional file 5: **Change from Baseline Body Weight, Lean Body Mass and Fat Mass (SD) for Completers by Gender.** (PDF 115 kb) Additional file 6: **Change from Baseline Body Weight, Lean Body Mass, and Fat Mass (SD) for Completers by Age Group.** (PDF 121 kb)

## Abbreviations

BMI: Body mass index
BPM: Beats per minute
CFR: Code of Federal Regulations
CI: Confidence interval
DBP: Diastolic blood pressure
DXA: Dual energy X-ray absorptiometry
HC: Hip circumference
ITT: Intention-to-treat
LOCF: Last observation carried forward
MWCC: Medifast Weight Control Center
PHI: Personal health information
SBP: Systolic blood pressure
SD: Standard deviation
TEE: Total energy expenditure
WC: Waist circumference
WHR: Waist-to-Hip ratio
