# Supplementary material for: Effectiveness of a Medifast meal replacement program on weight, body composition and cardiometabolic risk factors in overweight and obese adults: a multicenter systematic retrospective chart review study
Source: Nutr J. 2015 Aug 6;14:77. doi: 10.1186/s12937-015-0062-8 (PMC4527127; doi:10.1186/s12937-015-0062-8)
Supplement: Additional file 1: — Absolute and Percent Weight Change - 4 & 2 & 1 Plan – Completers Analysis. (PDF 38 kb) [file 12937_2015_62_MOESM1_ESM.pdf]

Absolute and Percent Weight Change - 4 & 2 & 1 Plan – Completers Analysis

Week 0 (Baseline)		*kg*		***Follow-up***	*kg*		*Within Group*	
	n	*mean*	*stdev*	***Visits***	*mean*	*stdev*	*Z*	*P value*
All Week 0	309	109.1	21.7					
Paired Sample Weeks 0 to 1	283	108.8	21.2	Week 1	106.1	20.6	-14.483	<0.0001
				Change: Week 0 to 1	-2.7	1.5		
				% Change	-2.47	1.25		
Paired Sample Weeks 0 to 2	280	109.1	22.1	Week 2	105.4	21.3	-14.498	<0.0001
				Change: Week 0 to 2	-3.7	1.9		
				% Change	-3.41	1.5		
Paired Sample Weeks 0 to 4	289	108.6	21.1	Week 4	103.1	19.8	-14.732	<0.0001
				Change: Week 0 to 4	-5.6	2.7		
				% Change	-5.08	2.13		
Paired Sample Weeks 0 to 8	239	108.6	21.3	Week 8	100.3	19.7	-13.402	<0.0001
				Change: Week 0 to 8	-8.3	4.2		
				% Change	-7.62	3.31		
Paired Sample Weeks 0 to 12*	185	107.6	21.1	Week 12	96.7	19.2	-11.795	<0.0001
				Change: Week 0 to 12	-10.9	5.6		
				% Change	-10.05	4.36		
Paired Sample Weeks 0 to 16	151	108.8	21.4	Week 16	95.7	19.0	-10.660	<0.0001
				Change Week 0 to 16	-13.1	6.6		
				% Change	-11.92	4.99		
Paired Sample Weeks 0 to 20	112	110.0	20.6	Week 20	95.2	18.1	-9.186	<0.0001
				Change Week 0 to 20	-14.7	7.4		
				% Change	-13.25	5.48		
Paired Sample Weeks 0 to 24	81	111.3	20.5	Week 24	95.3	18.2	-7.818	<0.0001
				Change Week 0 to 24	-16.0	7.9		
				% Change	-14.28	5.94		
Paired Sample Weeks 0 to FV	306	109.1	21.8	Week FV	98.5	20.8	-15.124	<0.0001
				Change Week 0 to FV	-10.6	8.5		
				% Change	-9.62	6.95		

*Primary Analysis

Completers included all individuals with weight data at the given visit. Final Visit (FV) represents an individual's last visit to the MWCC while on the 4 & 2 & 1 Plan. Significance levels are for within group changes from baseline body weight using Wilcoxon signed-rank tests.
