# Supplementary material for: Effectiveness of a Medifast meal replacement program on weight, body composition and cardiometabolic risk factors in overweight and obese adults: a multicenter systematic retrospective chart review study
Source: Nutr J. 2015 Aug 6;14:77. doi: 10.1186/s12937-015-0062-8 (PMC4527127; doi:10.1186/s12937-015-0062-8)
Supplement: Additional file 2: — Average Weekly Rate of Weight Loss by Time – 4 & 2 &1 Plan. (PDF 51 kb) [file 12937_2015_62_MOESM2_ESM.pdf]

Average Weekly Rate of Weight Loss by Time – 4 & 2 &1 Plan

Time Interval	Rate of Weight Change (kg/week)	95% CI
0 to 1 week	-2.71	(-2.87, -2.55)*
0 to 2 weeks	-1.89	(-1.99, -1.79)*
0 to 4 weeks	-1.36	(-1.41, -1.31)*
0 to 8 weeks	-1.00	(-1.03, -0.97)*
0 to 12 weeks	-0.84	(-0.86, -0.82)*
0 to 16 weeks	-0.75	(-0.77, -0.74)*
0 to 20 weeks	-0.69	(-0.70, -0.67)*
0 to 24 weeks	-0.63	(-0.65, -0.61)*

Random effect regression model estimating the association between weight change and time, assessed over different intervals while individuals were on the 4 & 2 &1 Plan; model included baseline weight as a covariate. * $p < 0.0001$.
