# Supplementary material for: Effectiveness of a Medifast meal replacement program on weight, body composition and cardiometabolic risk factors in overweight and obese adults: a multicenter systematic retrospective chart review study
Source: Nutr J. 2015 Aug 6;14:77. doi: 10.1186/s12937-015-0062-8 (PMC4527127; doi:10.1186/s12937-015-0062-8)
Supplement: Additional file 3: — Body Weight – Intention-to-Treat Last Observation Carried Forward - 4 & 2 & 1. (PDF 42 kb) [file 12937_2015_62_MOESM3_ESM.pdf]

Body Weight – Intention-to-Treat Last Observation Carried Forward - 4 & 2 & 1

Week 0 (Baseline)	*kgs*			***Follow-Up Visits***	*kg*			*Within Group Comparison**	
	n	*mean*	*stdev*		*n*	*mean*	*stdev*	*Z*	*P*
All Week 0	309	109.1	21.7						
Paired Sample Weeks 0 to 1	309	109.1	21.7	Week 1	309	106.6	21.3	-14.483	<0.0001
				Change: Week 0 to 1	309	-2.5	1.6		
				% Change		-2.27	1.38		
Paired Sample Weeks 0 to 2	309	109.1	21.7	Week 2	309	105.5	20.9	-15.124	<0.0001
				Change: Week 0 to 2	309	-3.6	2.0		
				% Change		-3.29	1.55		
Paired Sample Weeks 0 to 4	309	109.1	21.7	Week 4	309	103.8	20.6	-15.156	<0.0001
				Change: Week 0 to 4	309	-5.3	2.8		
				% Change		-4.83	2.23		
Paired Sample Weeks 0 to 8	306	109.2	21.7	Week 8	306	101.8	20.4	-15.067	<0.0001
				Change: Week 0 to 8	306	-7.4	4.4		
				% Change		-6.74	3.58		
Paired Sample Weeks 0 to 12	281	111.1	21.3	Week 12	281	102.3	20.1	-14.455	<0.0001
				Change: Week 0 to 12	280	-8.9	5.6		
				% Change		-7.94	4.66		
Paired Sample Weeks 0 to 16	235	114.6	20.5	Week 16	235	104.3	19.6	-13.218	<0.0001
				Change Week 0 to 16	235	-10.3	7.0		
				% Change		-8.95	5.74		
Paired Sample Weeks 0 to 20	201	117.3	19.9	Week 20	201	106.4	19.4	-12.218	<0.0001
				Change Week 0 to 20	201	-10.8	7.8		
				% Change		-9.22	6.35		
Paired Sample Weeks 0 to 24	157	121.2	19.4	Week 24	157	109.7	19.0	-10.801	<0.0001
				Change Week 0 to 24	157	-11.5	8.6		
				% Change		-9.39	6.81		
Paired Sample Weeks 0 to FV	306	109.1	21.8	Week FV	306	98.5	20.8	-15.124	<0.0001
				Change Week 0 to FV	306	-10.6	8.5		
				% Change		-9.62	6.95		

Mean absolute and percent change from baseline weight for the intention-to-treat last observation carried forward analyses. If missing, imputed data were carried through from the last measured observation to each individual's last prescribed week of weight loss. Final Visit represents an individual's last visit to the MWCC while on the 4 & 2 & 1 Plan. Significance levels were calculated based on within group changes from baseline body weight using Wilcoxon signed-rank tests.
