# Supplementary material for: Effectiveness of a Medifast meal replacement program on weight, body composition and cardiometabolic risk factors in overweight and obese adults: a multicenter systematic retrospective chart review study
Source: Nutr J. 2015 Aug 6;14:77. doi: 10.1186/s12937-015-0062-8 (PMC4527127; doi:10.1186/s12937-015-0062-8)
Supplement: Additional file 4: — Percent Achieving Specified Weight Loss – ITT LOCF. (PDF 73 kb) [file 12937_2015_62_MOESM4_ESM.pdf]

Percent Achieving Specified Weight Loss – ITT LOCF

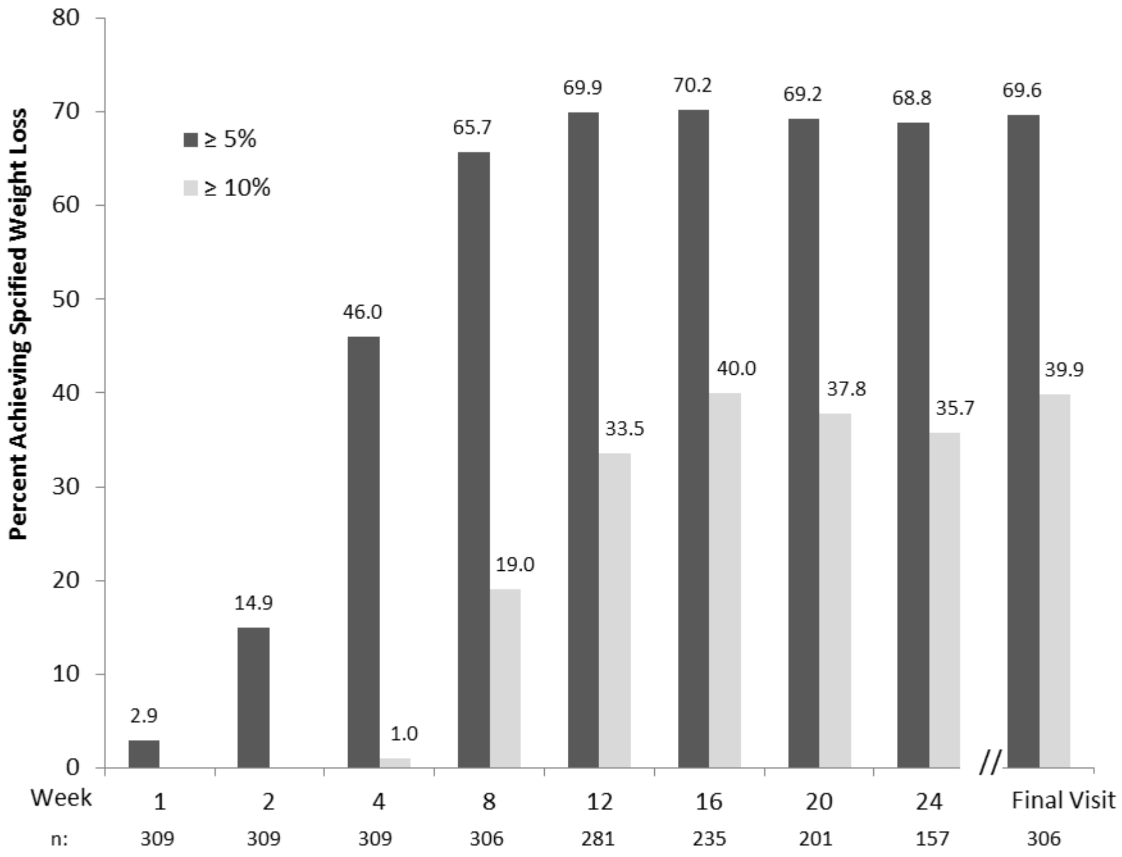

Intention-to-Treat Last Observation Carried Forward (ITT LOCF) values are shown; sample sizes are designated below the graph. Final Visit represents an individual's last visit to the MWCC while on the 4 & 2 & 1 Plan.
