# Supplementary material for: Effectiveness of a Medifast meal replacement program on weight, body composition and cardiometabolic risk factors in overweight and obese adults: a multicenter systematic retrospective chart review study
Source: Nutr J. 2015 Aug 6;14:77. doi: 10.1186/s12937-015-0062-8 (PMC4527127; doi:10.1186/s12937-015-0062-8)
Supplement: Additional file 5: — Change from Baseline Body Weight, Lean Body Mass and Fat Mass (SD) for Completers by Gender. (PDF 115 kb) [file 12937_2015_62_MOESM5_ESM.pdf]

Change from Baseline Body Weight (SD) for Completers by Gender

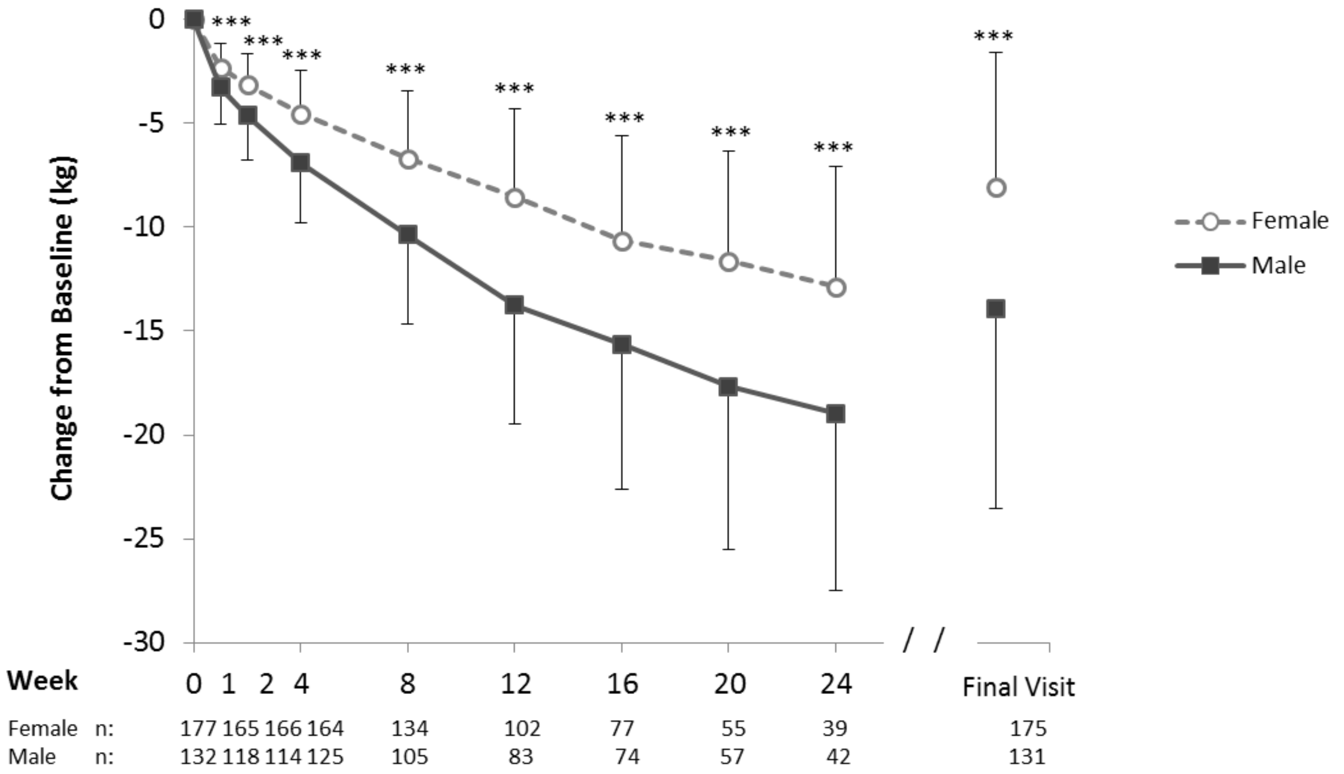

Mean (±SD) change from baseline body weight by gender for the Completers population which included all individuals with weight data at the given visit; sample sizes are designated below the graph. Final Visit represents an individual's last visit to the MWCC while on the 4 & 2 & 1 Plan. Significance level for within group changes from baseline were $p<0.0001$ for males and $p\leq0.001$ for females. Significance levels for between group comparisons using bivariate t-tests at each time point are shown: ***$p<0.0001$.

Change from Baseline Lean Body Mass and Fat Mass (SD) for Completers by Gender

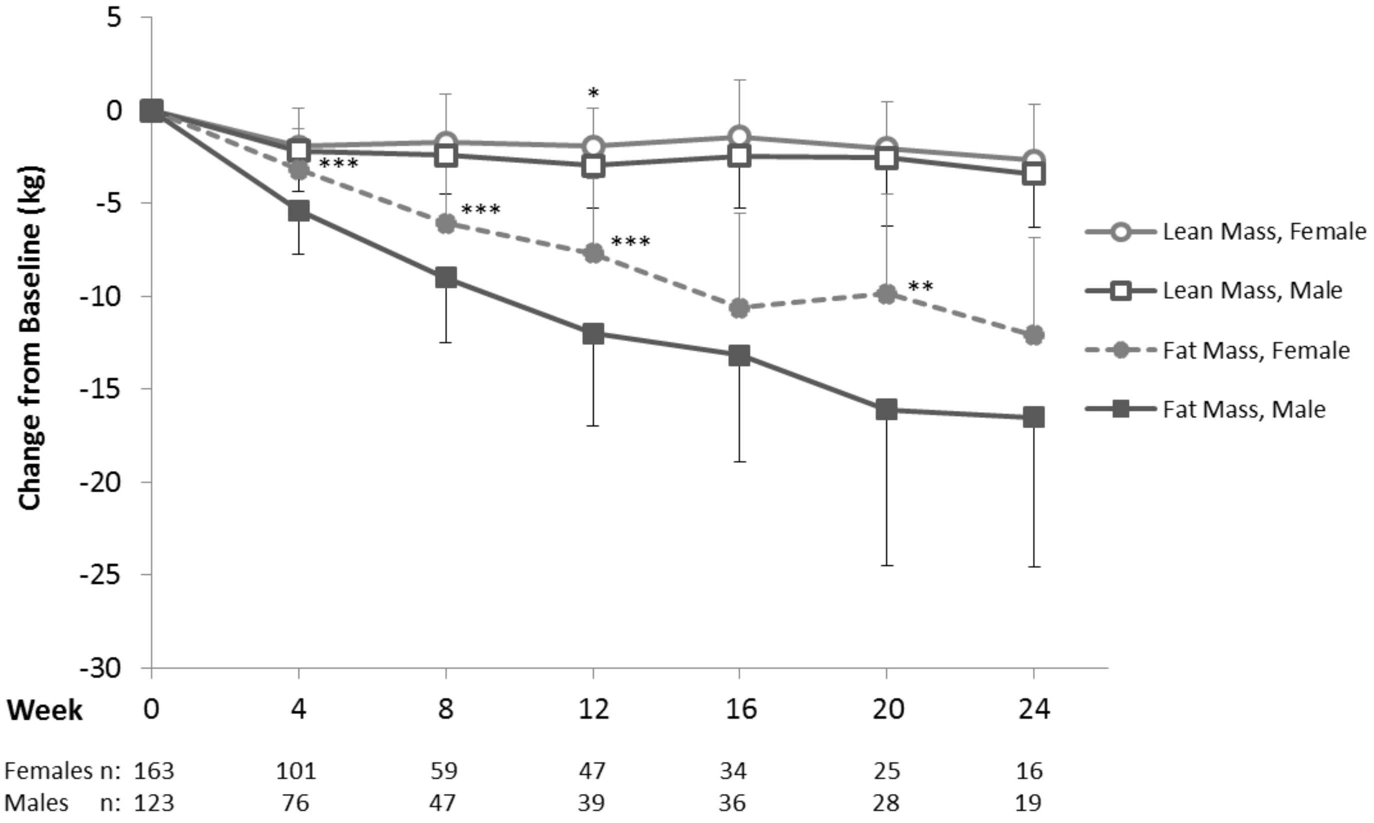

Mean (±SD) change from baseline lean body mass and body fat mass for Completers by gender. The Completers population included all individuals with weight data at the given visit; sample sizes are designated below the graph. Significance level for all within group changes from baseline were $p<0.0001$ for fat mass and $p<0.01$ for lean mass in both genders at all time points. Significance levels for between group comparisons using bivariate t-tests at each time point are shown: *$p<0.05$; **$p<0.01$; ***$p<0.0001$.
